# Supplementary material for: scRNAseq and High-Throughput Spatial Analysis of Tumor and Normal Microenvironment in Solid Tumors Reveal a Possible Origin of Circulating Tumor Hybrid Cells
Source: Cancers (Basel). 2024 Apr 8;16(7):1444. doi: 10.3390/cancers16071444 (PMC11010995; doi:10.3390/cancers16071444)

**Supplementary Figure 1**:

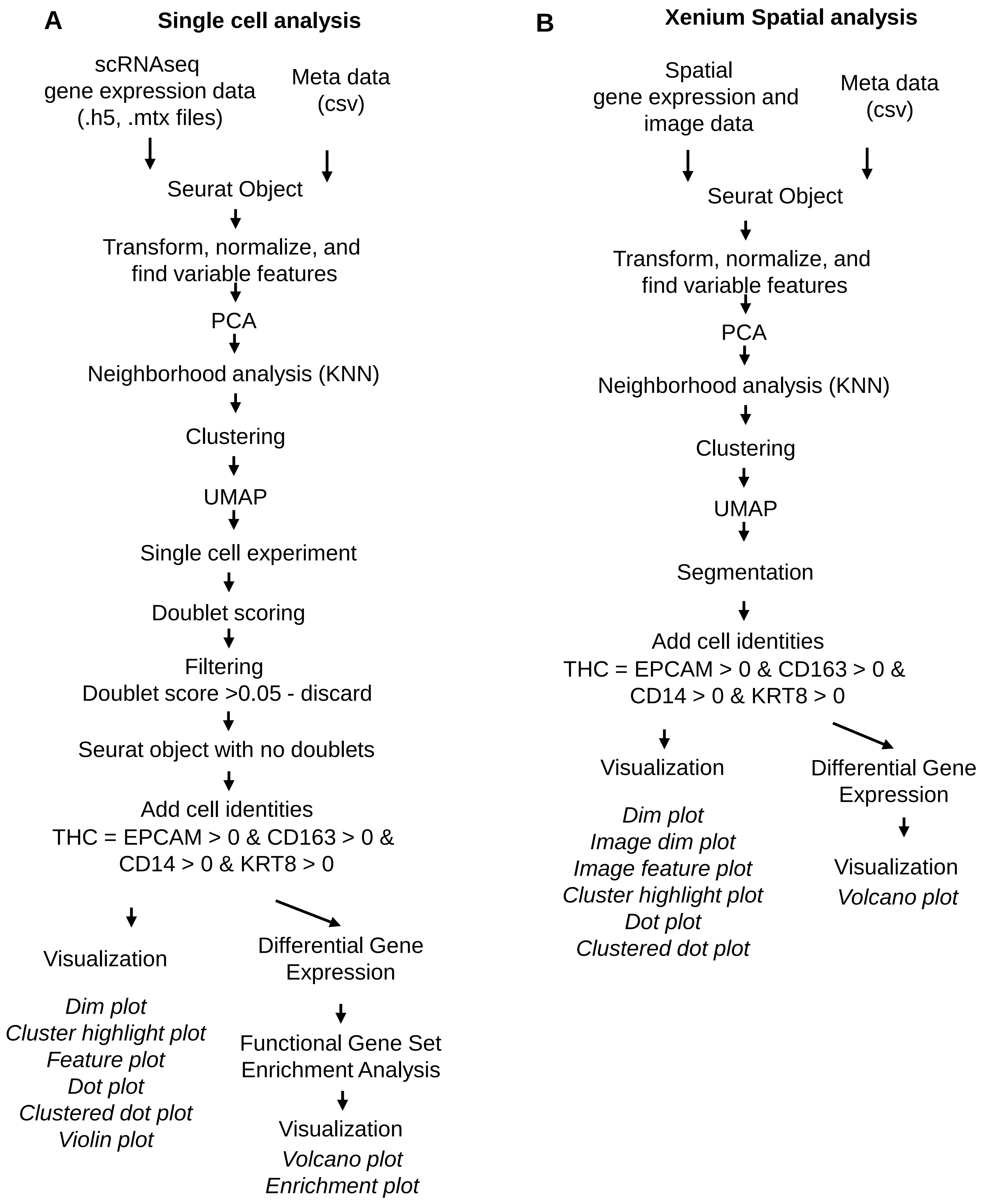

**Supplementary Figure 2**:

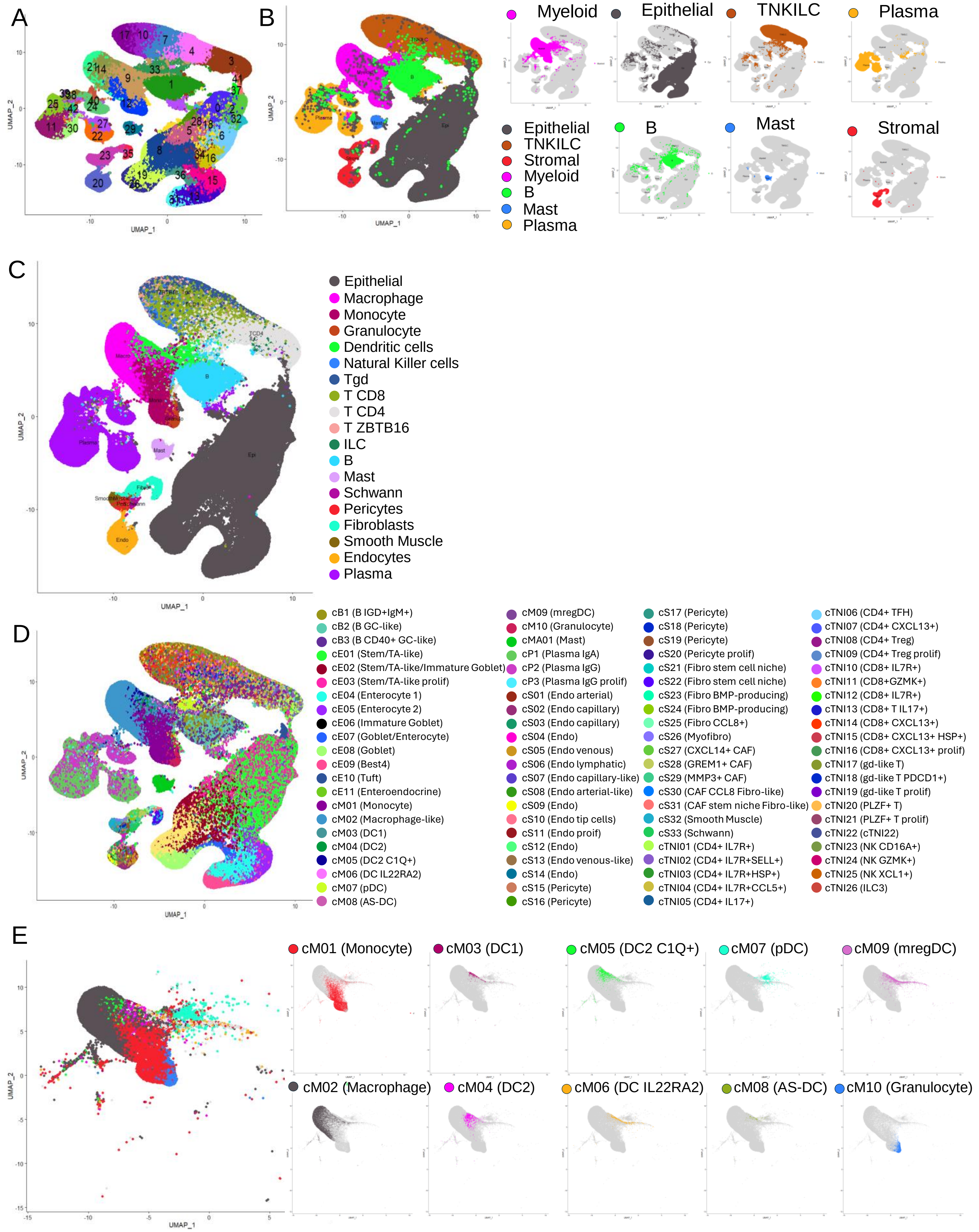

**Supplementary Figure 3**:

A

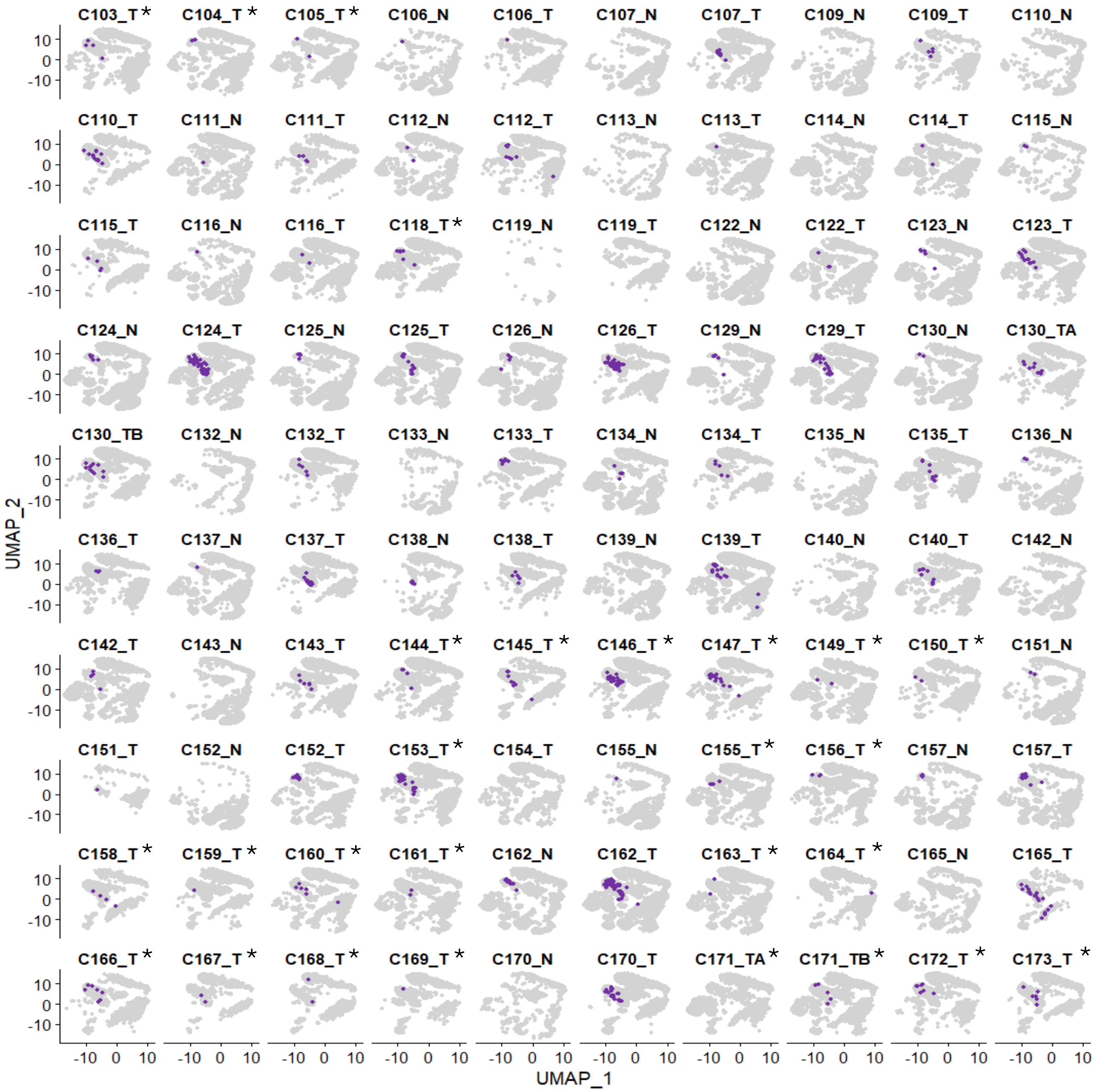


B

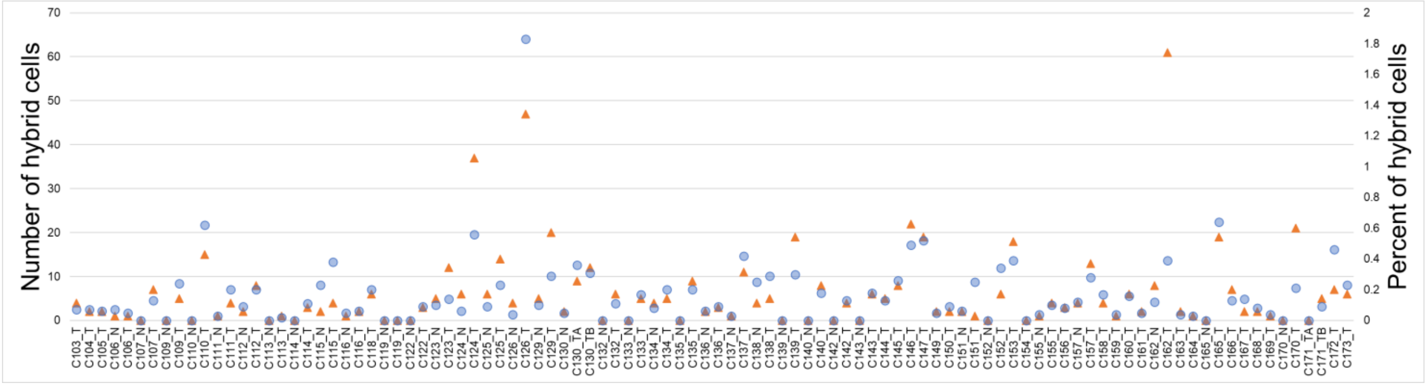

**Supplementary Figure 4**:

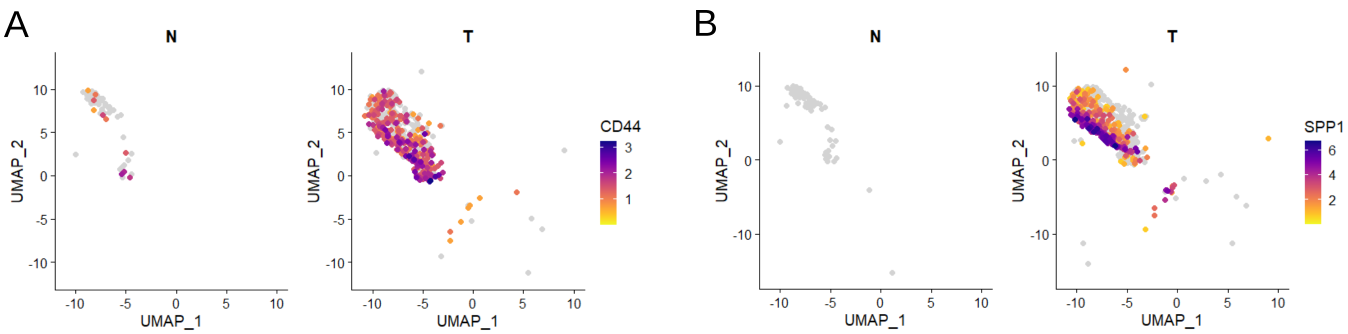

**Supplementary Figure 5:**

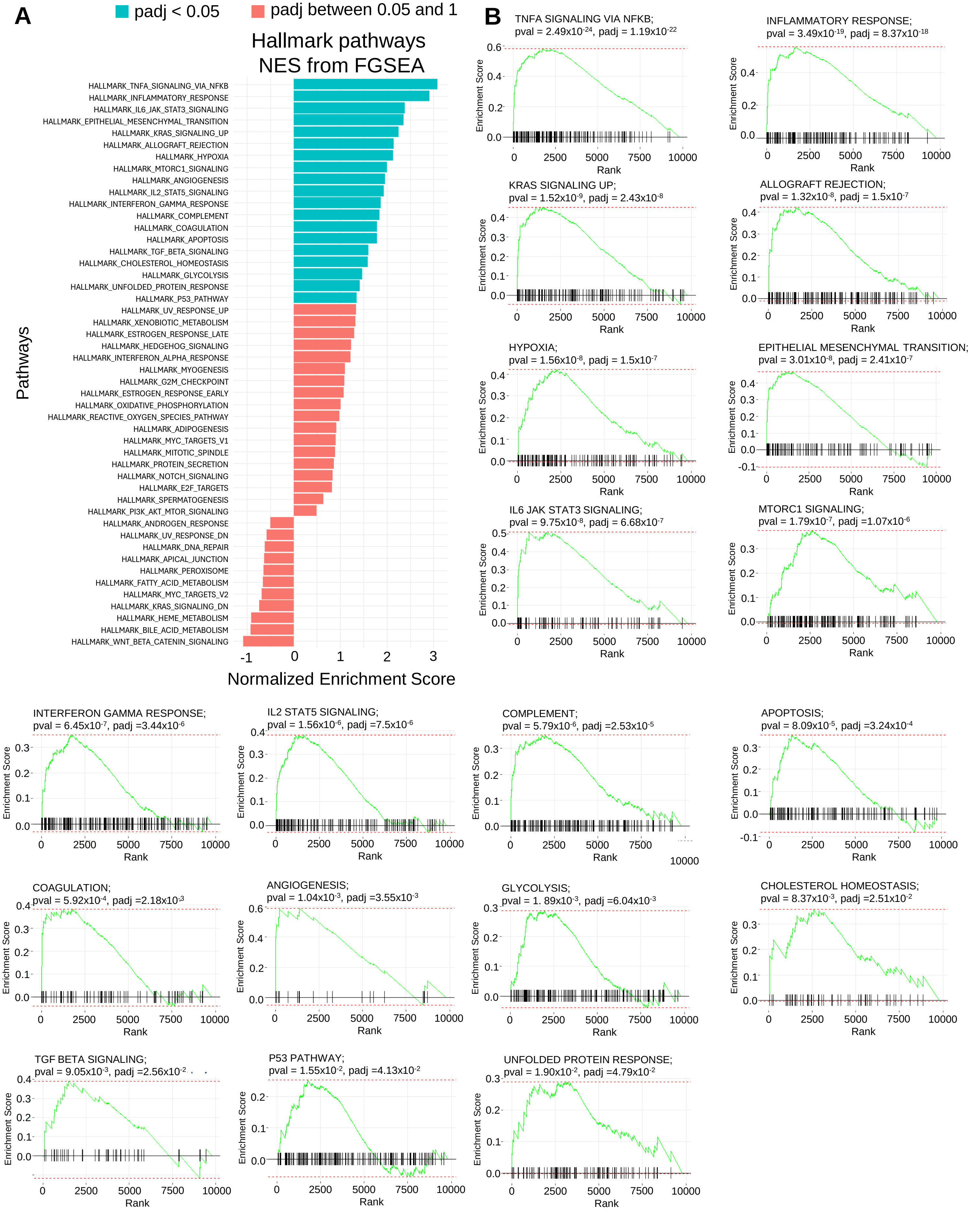

**Supplementary Figure 6**:

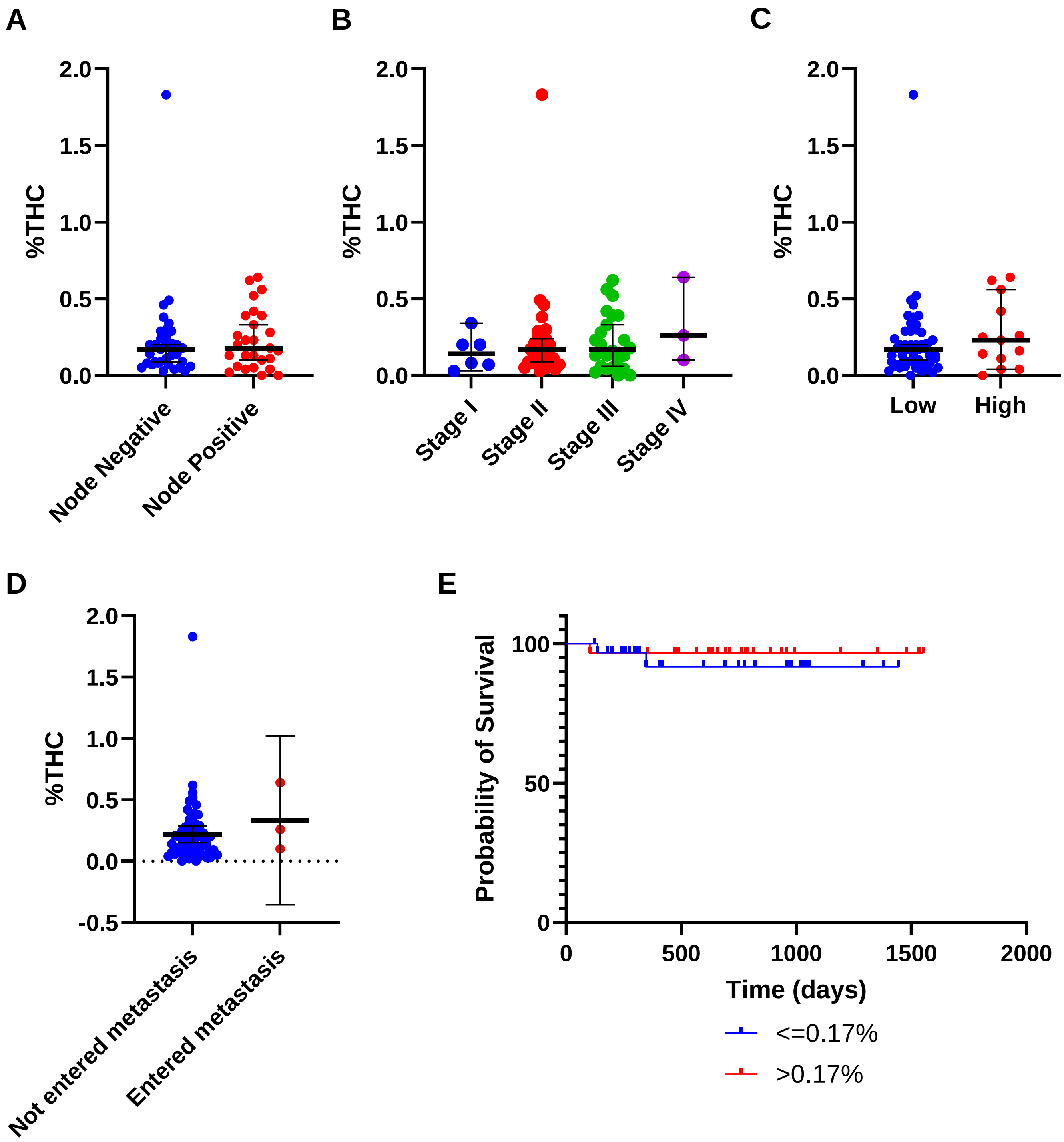

**Supplementary Figure 7**:

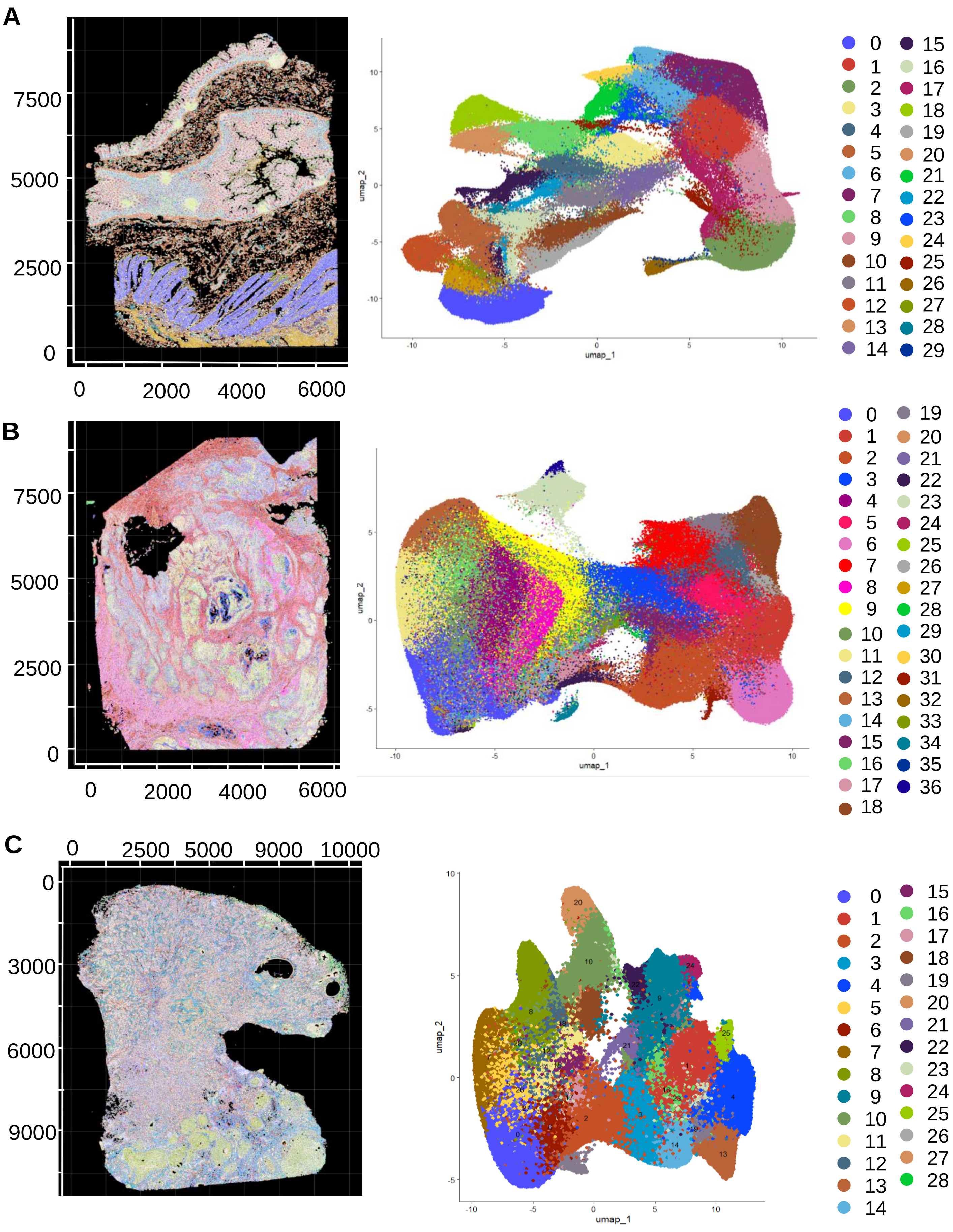

**Supplementary Figure 8**:

**A** Normal colon

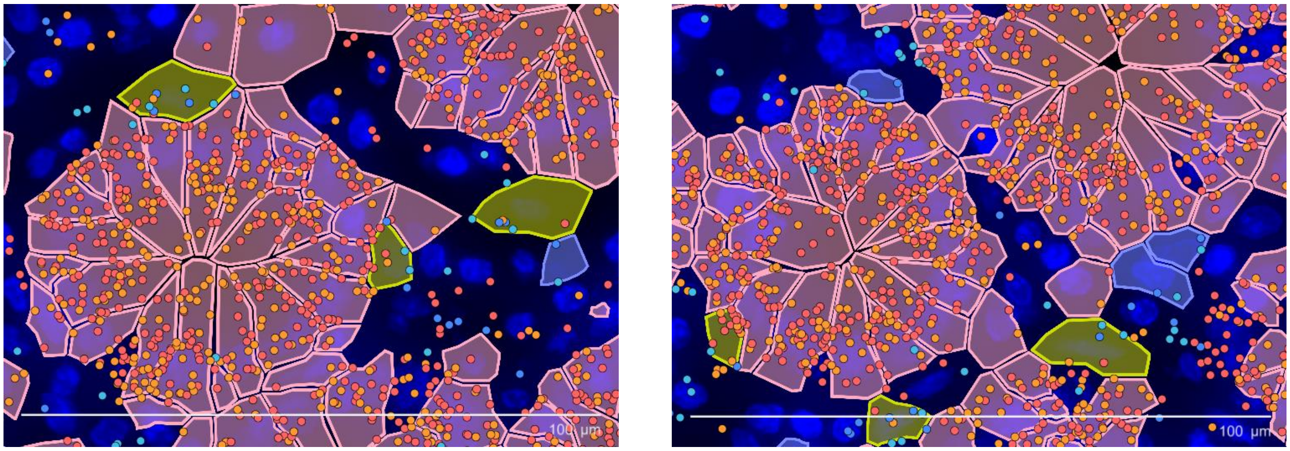


**B** Tumor colon

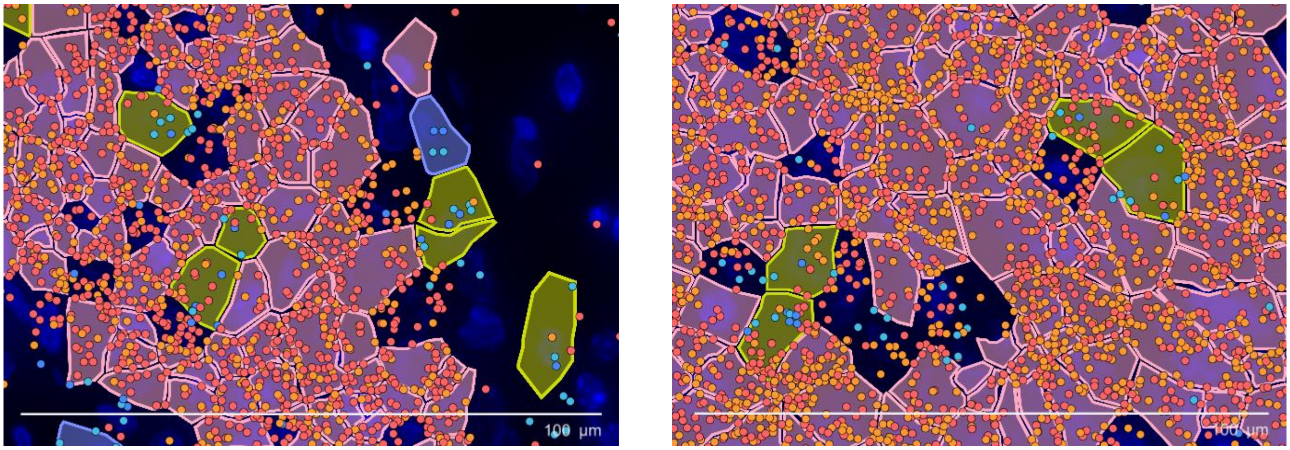


**C** Tumor breast

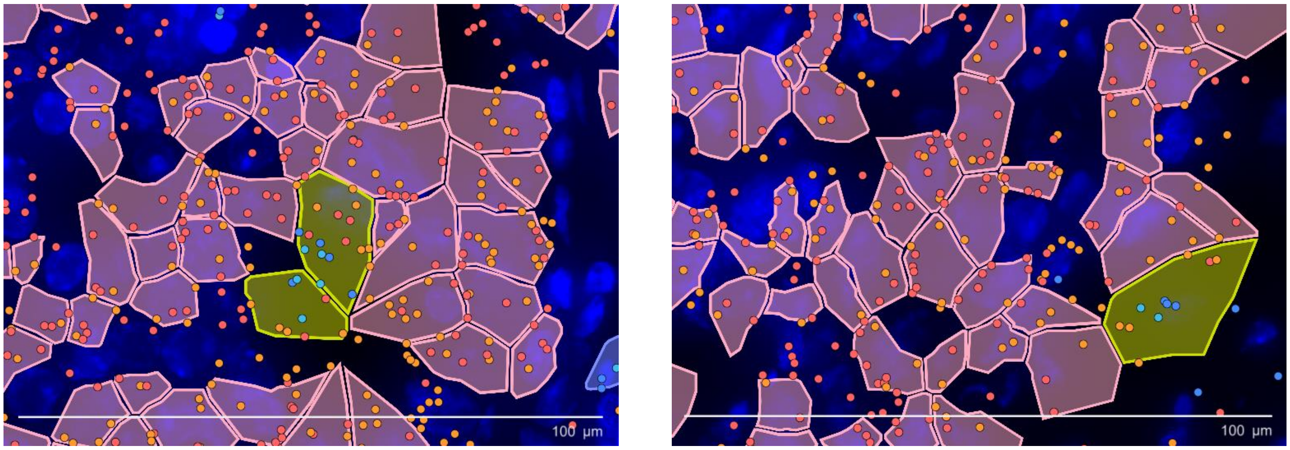


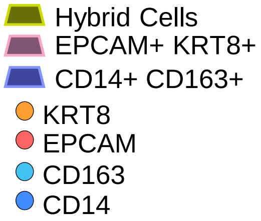

**Supplementary Figure 9**:

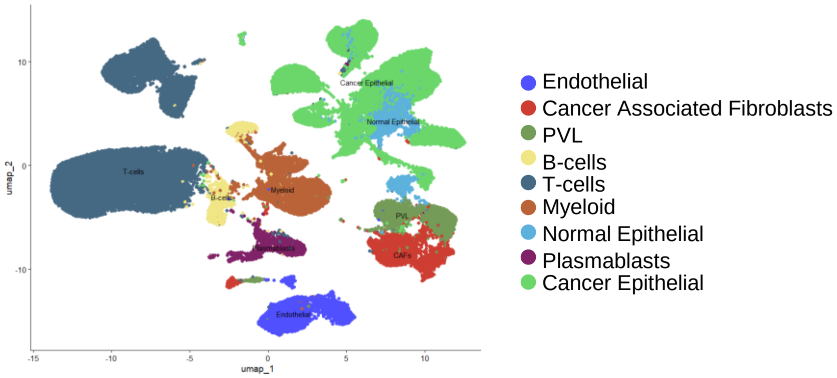

**Supplementary Figure 10**:

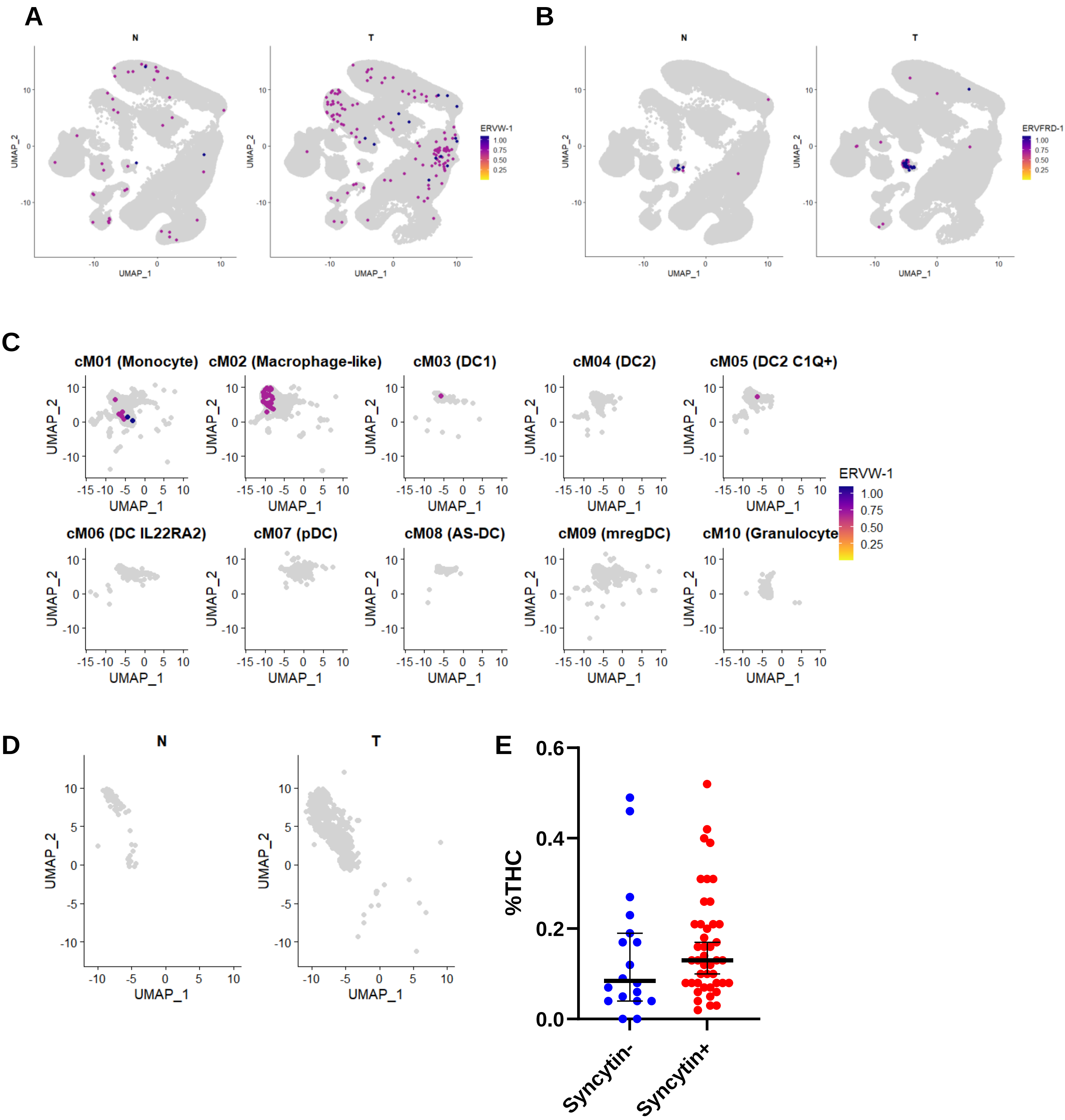

Supplement: Supplementary file 1 [file cancers-16-01444-s001.zip › Figures_Final_Supp_R.pdf]
